# Dietary intake of polybrominated diphenyl ethers (PBDEs) and the risk of obesity in the French E3N cohort

**DOI:** 10.1038/s41366-026-02109-z

**Published:** 2026-06-02

**Authors:** Xuan Ren, Pauline Frenoy, Fanny Artaud, Chloé Marques, Gianluca Severi, Geneviève Nicolas, Inge Huybrechts, Francesca Romana Mancini

**Affiliations:** 1 UVSQ, Inserm, CESP, Gustave Roussy, Université Paris-Saclay, Villejuif, France; 2 Department of Statistics, Computer Science, Applications “G. Parenti”, University of Florence, Florence, Italy; 3 Nutrition and Metabolism Branch, International Agency for Research on Cancer, Lyon, France

**Keywords:** Obesity, Risk factors

## Abstract

**Background:**

Polybrominated diphenyl ethers (PBDEs) are persistent organic pollutants, with the primary exposure pathway for the general population being the consumption of contaminated food. Human exposure to PBDEs is suspected to increase the risk of obesity.

**Objectives:**

This study aims to investigate the association between intake of PBDEs and the risk of obesity/overweight or weight gain in the French Etude Epidémiologique auprès des femmes de l’Education Nationale (E3N) cohort.

**Methods:**

This study included 66 467 women with a mean age of 52.47 years over a 20-years follow-up. Cox proportional hazard regression models estimated hazard ratios (HRs) and 95% confidence intervals (CIs) for the associations between PBDEs intake and risk of obesity, overweight and gain more than 10 kg.

**Results:**

Higher PBDEs intakes were related to increased risk of overweight (HR (1-SD increment) = 1.07 (1.05–1.09), HR (Q4 vs. Q1) = 1.13 (1.07–1.19), *P*-trend <0.001) and weight gain (HR (1-SD increment) = 1.12 (1.09–1.14), HR (Q4vsQ1) = 1.28 (1.21–1.37), *P*-trend <0.001). A non-linear association (*P*-non-linearity = 0.0014, *P*-overall <0.001) between the intake of PBDEs and obesity risk (HR (Q4 vs. Q1) = 1.25 (1.15–1.37), *P*-trend <0.001) was identified. The associations remained consistent when adjusting in addition separately for total fat, polyunsaturated fatty acids (PUFA), n-3 PUFA, the main food sources of PBDEs intake as well as when running stratified analyses based on the follow-up duration.

**Conclusions:**

This study identified a positive association between intake of PBDEs and the risk of overweight, obesity and weight gain. Further efforts are needed to reduce PBDEs contamination in food and lower exposure levels in the general population.

## Introduction

Obesity is traditionally defined as a body mass index (BMI) above 30 kg/m^2^, while being overweight is defined as a BMI between 25 and 30 kg/m^2^. A World Health Organization (WHO) report published in 2022 revealed that 60% of the European population are people with overweight or obesity, highlighting the severity of this public health issue [1]. In France, 47.3% of individuals were living with overweight or obesity in 2020, with the prevalence of overweight remaining stable at around 30% since 1997, while the prevalence of obesity has risen from 8.5% in 1997 to 17% in 2020 [2]. Notably, the prevalence of overweight and obesity is higher among older adults, with 57.3% of those aged 65 and over affected, compared to 23.2% of individuals aged 18–24. Moreover, men were more likely to be overweight than women (36.9% vs. 23.9%), but women had a slightly higher prevalence of obesity (17.4% vs. 16.7%) [2]. Previous studies have shown that excessive fat accumulation and weight gain are associated with an increased risk of cardiovascular disease, several types of cancer, and other adverse health outcomes. A weight gain of 10 kg has been used as a threshold to characterise clinically meaningful weight gain [3–6].

Accumulating evidence suggests that the rising prevalence of obesity in the last few decades may be partly due to exposure to persistent organic pollutants (POPs) [7]. The European Commission has declared POPs to be a threat to both the global environment and human health. Certain POPs are known or suspected to act as endocrine disrupting chemicals (EDCs) due to their ability to disrupt the endocrine system and, in turn, also predispose people to gain excessive weight [7, 8].

Among these EDCs are polybrominated diphenyl ethers (PBDEs)—a group of substances with theoretically 209 congeners. Out of the 209 congeners, evidence has shown concern for at least 8 of them: BDE-28, −47, −99, −100, −153, −154, −183 and −209 [9]. Between the 1970s and the early 2000s, PBDEs were widely used in household furnishings, textiles, building materials and electronics. However, their usage has been increasingly regulated due to recognised toxicity [10]. Despite regulations, PBDEs remain of significant public health concern because of their resistance to degradation and ability to accumulate in adipose tissue. Humans can be exposed to PBDEs through inhalation, diet, and maternal transfer from mother to child [11]. Several studies have suggested that diet represents a major pathway of PBDEs exposure for non-occupationally exposed individuals [12–14]. As a result, the general population continue to be exposed to PBDEs particularly through the consumption of contaminated food, with the main food sources being fish, dairy products and meat [10]. In addition, if dietary PBDE exposure is associated with an increased risk of weight gain, this information could provide a basis for developing specific public health strategies aimed at reducing exposure and achieving meaningful health benefits. Human exposure to PBDEs has been reported to adversely affect glucose metabolism, thyroid, and ovarian function, potentially influencing weight change and the risk of obesity [15]. Several studies have evaluated the associations between prenatal PBDE levels and their effects on birth weight and childhood obesity, reporting reduced birth weight and inverse associations with body fat in childhood [16, 17]. Only a few prospective studies have investigated the relationship between blood PBDE levels and obesity in adults, and the results have been inconsistent. Some studies reported no association [18, 19] or inverse association [18]. while others observed positive associations [20–22]. Nevertheless, previous studies had relatively small sample sizes with short follow-up duration, and mainly focused on a few specific PBDE congeners measured in blood and specific populations. Due to these inconsistent results and the limited number of studies among adults, further research with large sample sizes and longer follow-up durations is necessary to assess the associations between dietary intake of PBDEs and the risk of obesity or weight gain.

Therefore, the main objective of the present study was to investigate the association between dietary intake of PBDEs and the risk of obesity/overweight or weight gain in the French *Etude Epidémiologique auprès des femmes de l’Education Nationale* (E3N) prospective cohort study.

## Materials and methods

### Study population

The E3N study is a large ongoing prospective cohort study involving 98,995 French women born between 1925 and 1950, primarily insured through the *Mutuelle Générale de l’Education Nationale* (MGEN). Recruitment began in 1990 with a baseline self-administered questionnaire and informed consent, follow-up questionnaires were dispatched every 2–3 years. The average response rate was approximately 83%, with an overall loss to follow-up of only 3% since 1990. The study was approved by the French National Commission for Data Protection and Privacy [23].

Between 1993 and 2018, detailed information was collected at various time points, including anthropometric characteristics, health status, diet, reproductive history, hormonal treatments, smoking habits, alcohol consumption, or physical activity [23].

**Fig. 1:**
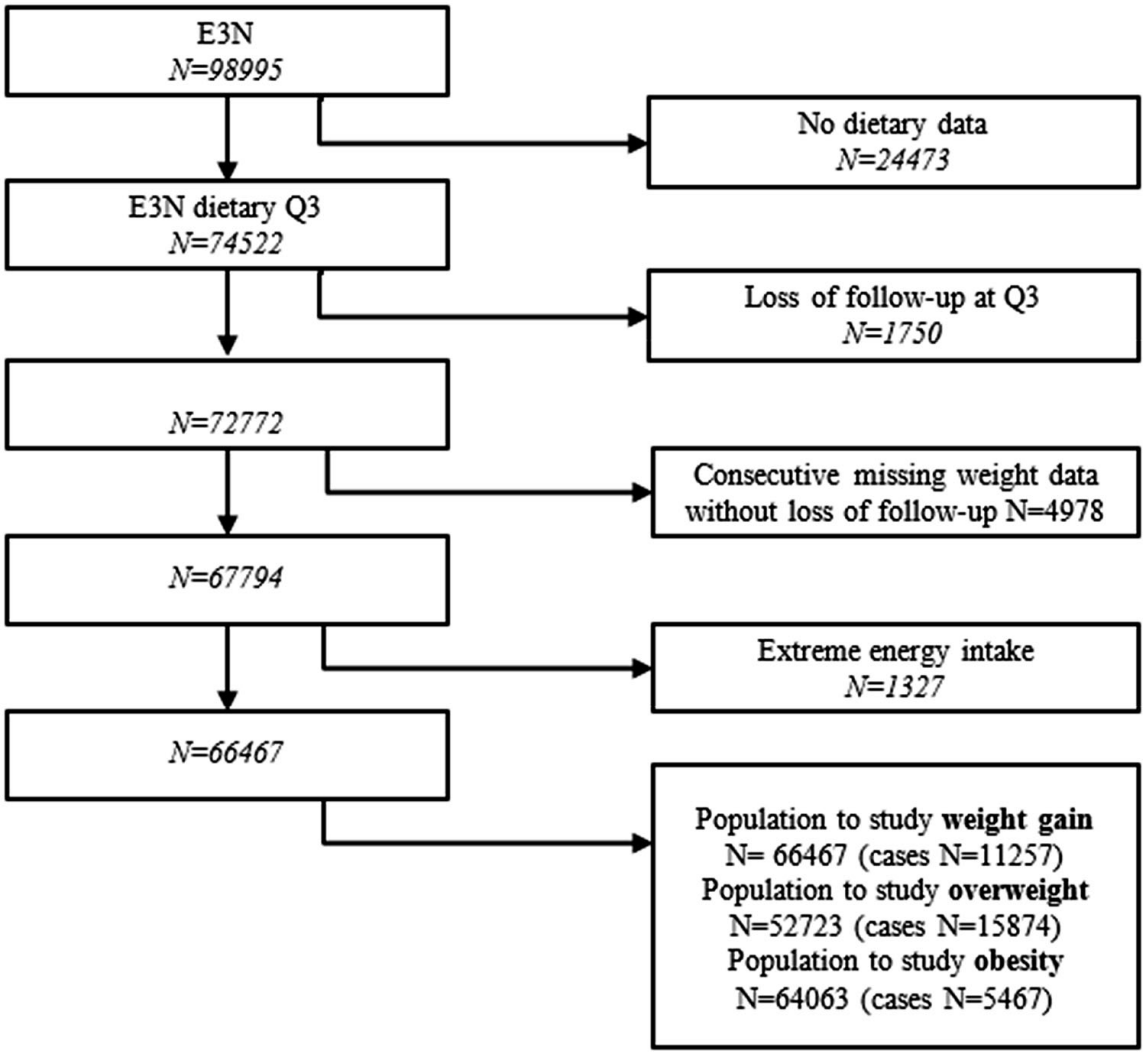
Study population.

For this study, we included 74,522 women who completed the dietary questionnaire in 1993 (Q3, baseline for the present study). The E3N cohort consisted of middle-aged women with a median age of 51 in 1993, making the inclusion of pregnant women highly unlikely due to their age and the timeframe of the study. Participants were excluded if follow-up ended at Q3, if baseline height or weight was missing, or if weight data were missing on consecutive questionnaires. To avoid under or over reporting of dietary intake, 1327 participants in the top or bottom 1% of the ratio of energy intake to energy requirement were excluded [24, 25]. Finally, to assess the risk of obesity and overweight, participants with prevalent obesity or overweight at baseline were excluded. Consequently, the study population size varied depending on the case variable considered (obesity, overweight, or weight gain of more than 10 kg) (Fig. 1).

### Assessment of food consumption

Dietary data were estimated in 1993 using a validated semi-quantitative food frequency questionnaire, which contained 208 food items. The dietary information collected included details on breakfast, morning snack, aperitif before lunch, lunch, afternoon snack, pre-dinner aperitif, dinner, and after dinner snack. Women’s daily intake of nutrients, such as fat intake, was then calculated based on data from the French Information Center on Food Quality (CIQUAL) [26]. The validity and reproducibility of the dietary questionnaire have been tested and previously described [27].

### Assessment of dietary intake of PBDEs

Data on PBDEs food contamination were obtained from the 2nd French Total Diet Study (TDS2) conducted by the French Agency for Food, Environmental and Occupational Health and Safety (ANSES) [5, 6, 28, 29]. Each food sample analysed in TDS2 was categorised and described according to the nomenclature used in the French national food consumption study INCA2 (Étude Individuelle Nationale des Consommations Alimentaires 2) and that PBDE concentration values for each sample were reported individually. The TDS2 database is open source [30]. A total of approximately 20,000 food products were collected across eight French regions between June 2007 and January 2009 and combined into 1352 composite samples representative of typical French dietary habits. Eight PBDEs were measured in food corresponding to the main known sources of exposure, which include meat, fish, eggs and dairy products [31]. In the present study, PBDE values in food items below the limit of detection were replaced by 0 (lower-bound scenario). The E3N database for food consumption and the ANSES database for food contaminant concentrations were subsequently merged, as described elsewhere [32]. The daily mean dietary intake of each PBDE (BDE-28, BDE-47, BDE-99, BDE-100, BDE-153, BDE-154, BDE-183 and BDE-209; ng/day) was estimated by multiplying the mean daily quantities consumed of each food component by the values of contamination of the corresponding food component [10]. The intake of dioxins and polychlorinated biphenyls (PCBs) was collected and estimated using the same approach as for PBDEs [33].

### Identification of obesity, overweight and weight gain

In the E3N cohort, height and weight were self-reported by participants at each E3N questionnaire. The BMI is defined as the body weight divided by the square of the body height (kg/m^2^) [33]. A validation study involving 152 women from the Paris center of the cohort found strong correlations between self-reported and technician-measured anthropometric factors, with coefficients of 0.94 for weight and 0.92 for BMI [34].

In the present study,

- Obesity: is defined as BMI ≥ 30 kg/m ^2^ [35].
- Overweight: is defined as BMI ≥ 25 kg/m ^2^ [35].
- Weight gain: is defined as weight gain of more than 10 kg compared with the weight reported at baseline

### Covariates

Adjustment variables included in the analyses described below were defined by Directed Acyclic Graph (DAG) (supplementary Fig. 1) in order to assess the total effect of PBDEs intake on the risk of obesity, overweight or weight gain.

Information on educational level (duration <12 years; 12–14 years; >14 years) was collected at the first questionnaire sent in 1991 (Q1). Information on smoking status (non-smoker; former smoker; current smoker), parity (nulliparous; one or two children; more than 3 children), silhouette at puberty (very thin, thin, medium, wide, very wide), menopausal status and recent use of menopausal hormone therapy (MHT) (pre-menopaused, menopaused with recent use of MHT, i.e. less than a year ago; menopaused without recent use of MHT, no information) and utilisation of contraceptive pill (ever/never) was obtained from the second questionnaire sent in 1992 (Q2). In addition, information on physical activity (continuous, in metabolic equivalents of task (MET)-hour/week), daily intake of alcohol (continuous, in g/day), daily intake of lipids (continuous, in g/day), daily intake of fatty acids (continuous, in g/day) and daily total energy intake (continuous, in kcal/day) was obtained from the dietary and non-dietary questionnaires sent in 1993 (Q3). The Programme National Nutrition Santé (PNNS) adequacy score is a composite dietary index based on 13 components, including seven adequacy components (fruits and vegetables, nuts, legumes, whole-grain foods, milk and dairy products, fish and seafood, and added fats) and six moderation components (red meat, processed meat, sugary foods, sweet-tasting beverages, alcoholic beverages, and salt), reflecting adherence to French dietary recommendations. A low score, indicating poor adherence to recommendations, has been associated with increased risk of mortality and type 2 diabetes [36].

The modal value (the most frequently occurring value, for categorical variables) or the median (for continuous variables) was used to impute missing values for covariates with less than 5% missing data, in order to reflect the most common or central values in the population. In the present study, missing data for education level (3.4%) were imputed as 12–14 years; missing data for parity (0.7%) were imputed as one or two children; missing data for contraceptive pill use (0.5%) were imputed as ever use; missing data for silhouette at puberty (3.7%) were imputed as thin; and missing data for smoking status (0.8%) were imputed as never smoking. Missing data for physical activity (0.6%) were imputed using the median value within the entire population (37.97 MET-hours/week).

### Statistical analyses

Participants’ baseline characteristics were described according to quartile groups of total PBDE intakes, and separately among cases and non-cases. Also, the proportion of each PBDE congener in the total dietary intake of PBDEs in the study population and the correlations between each PBDE were described.

Cox proportional hazard regression models were used to estimate hazard ratios (HRs) and 95% confidence intervals (95% CI) for associations between the dietary intake of PBDEs and the risk of obesity, overweight and more than 10 kg weight gain. Adjustment variables included in the models were selected using the DAG (supplementary Fig. 1). For Cox proportional hazards models, the proportional hazards assumption was assessed using Schoenfeld residuals and no significant violations were detected. All models were adjusted on age as time scale. Age at exit was the age at which the participant developed obesity (or overweight or gained 10 kg), or the age at the last completed questionnaire before death or the end of follow-up; or age at the last completed questionnaire for lost to follow-up, whichever occurred first.

The first model was adjusted for age as time scale. Model 2 was further adjusted on education level, smoking status, silhouette at puberty, alcohol consumption (g/day), total energy intake without alcohol (kcal/day), and physical activity (MET-hours/week). Model 3, the main model, including all the variables identified by the DAG, was additionally adjusted on BMI at baseline, contraceptive pill use, parity and menopausal status and recent use of menopausal hormone therapy.

In the Cox model, dietary intake of total PBDEs was assessed both as continuous variables and as categorical variables into quartiles. The first quartile group for PBDEs dietary intake was used as reference. For the continuous exposure variables, standardisation was performed by dividing the intake of PBDEs by its respective standard deviation (SD), thereby estimating the HR per one SD increment. To assess linear trends across categories of PBDEs dietary intake, the median value was allocated to each category and subsequently employed as a continuous variable within the models.

Spline functions were used in model 3 to assess any non-linear association between PBDEs and risk of obesity, overweight or weight gain. Variables were modelled using Restricted Cubic Splines (RCS) with four knots (0.05th, 0.35th, 0.65th and 0.95th) [37]. Since BMI at baseline did not meet the assumptions of log-linearity and proportionality of hazards, it was included in the model by stratifying the baseline hazard function into quintiles of baseline BMI (20.40 kg/m^2^, 21.72 kg/m^2^, 23.11 kg/m^2^, 25.10 kg/m^2^). This approach allowed for accounting for BMI at baseline without making assumptions about its relationship with the outcome.

### Sensitivity analyses

Sensitivity analyses were performed adjusting model 3 for PNNS adequacy score, to disentangle the effects of exposure to food contaminants from those of overall quality of the diet. The total fat intake, polyunsaturated fatty acids (PUFA) intake, and n-3 polyunsaturated fatty acids (n-3 PUFA) intake were separately adjusted to distinguish the effect of exposure to food contaminants from those of fat intake (model 3). Model 3 was also adjusted for the sum of dioxins and dioxin-like polychlorinated biphenyls (Dioxins+DL-PCBs) and non-dioxin like PCBs (NDL-PCBs) to adjust for the effect of exposure to other food contaminants associated with the risk of weight gain [33]. In order to account for differences in energy intake, a residual contaminant model (adjusted for energy) was used based on Model 3. In this model, total PBDEs intakes were regressed on total energy intake, and the residuals from this regression were used as the intake variables [38]. Model 3 was additionally adjusted separately on the fish, dairy and meat consumption to investigate potential residual confounding from the diet. To minimise potential reverse causality and reduce the influence of early outcome-related dietary changes, we conducted a 5-year lag analysis. In this analysis, participants who developed obesity, became overweight, or gained more than 10 kg during the first five years of follow-up after Q3 were excluded (Model 3). The first five years were selected because dietary changes occurring shortly after baseline could be influenced by early disease-related weight changes, and excluding this period helps to better capture the temporal relationship between diet and subsequent weight outcomes.

### Subgroup analyses

Subgroup analyses were conducted based on the median follow-up time to evaluate long-term effects of PBDE dietary intake. In the first analysis, follow-up was stopped at the median, and in the second analysis, participants with follow-up periods shorter than median follow-up times were excluded. In order to evaluate potential effect modification, interaction analyses were performed between silhouette at puberty (very thin and thin, medium, large and very large) and PBDEs intake, as well as between baseline BMI and PBDEs intake. Subgroup analyses were performed only when the interaction tests were statistically significant.

All statistical analyses were performed with SAS software version 9.4 (SAS institute) and R version 4.3.1. All tests were two-sided and we considered *P* < 0.05 to be statistically significant.

## Results

### Characteristics of the study population

Among the 66,467 included participants, 11,257 women gained more than 10 kg during the follow-up (20.00 ± 6.62 years). Of the 52 723 women without overweight at baseline, 15,874 became overweight, and among the 64,063 without obesity at baseline, 5467 developed obesity during follow-up. Baseline characteristics of the overall study population and by quartile groups of PBDEs intakes are described in Table 1. The mean age at baseline was 52.47 years, and the mean BMI was 22.94 kg/m^2^, nearly 20% reporting having a large or very large silhouette at puberty. Most participants had more than 12 years of education, and more than half of the study population had never smoked. Baseline characteristics of cases and non-case participants were also described according to three outcomes (Supplementary Tables 1–3).

**Table 1:** Baseline characteristics of study participants from the E3N cohort for the overall population and by quartile groups of total PBDEs dietary intakes (mean ± SD or *N* (%)).

		Total PBDEs daily dietary intake (ng/day)			
	All (*N* = 66 467)	Q1 (*N* = 16 616)	Q2 (*N* = 16 617)	Q3 (*N* = 16 618)	Q4 (*N* = 16 616)
Age (years)	52.47 ± 6.68	53.75 ± 6.89	52.58 ± 6.70	51.86 ± 6.43	51.69 ± 6.48
Duration of the follow-up (years)	20.00 ± 6.62	19.90 ± 6.58	20.25 ± 6.47	20.11 ± 6.59	19.69 ± 6.88
Dietary intake (ng/day)					
Total PBDEs	41.99 ± 16.64	23.60 ± 5.22	35.12 ± 2.68	45.07 ± 3.20	64.15 ± 13.32
BDE-28	0.48 ± 0.34	0.26 ± 0.14	0.39 ± 0.19	0.52 ± 0.26	0.77 ± 0.46
BDE-47	8.21 ± 4.83	4.60 ± 1.91	6.80 ± 2.47	8.81 ± 3.38	12.64 ± 6.11
BDE-99	3.11 ± 1.19	1.96 ± 0.54	2.73 ± 0.56	3.34 ± 0.70	4.39 ± 1.20
BDE-100	1.65 ± 1.04	0.92 ± 0.43	1.36 ± 0.56	1.77 ± 0.76	2.57 ± 1.35
BDE-153	0.78 ± 0.31	0.48 ± 0.15	0.69 ± 0.16	0.85 ± 0.19	1.11 ± 0.30
BDE-154	0.88 ± 0.57	0.48 ± 0.23	0.72 ± 0.31	0.94 ± 0.41	1.37 ± 0.73
BDE-183	0.99 ± 0.43	0.63 ± 0.25	0.89 ± 0.27	1.07 ± 0.31	1.38 ± 0.45
BDE-209	25.88 ± 12.02	14.27 ± 4.15	21.55 ± 4.11	27.77 ± 5.46	39.93 ± 12.64
BMI (kg/m^2^)	22.94 ± 3.25	22.55 ± 3.06	22.76 ± 3.11	22.98 ± 3.20	23.46 ± 3.55
<18.5	2254 (3.39)	802 (4.83)	556 (3.35)	511 (3.07)	385 (2.32)
[18.5–25]	50,602 (76.13)	12,930 (77.82)	12,977 (78.09)	12,648 (76.11)	12,047 (72.50)
[25–30]	11,245 (16.92)	2469 (14.86)	2595 (15.62)	2917 (17.55)	3264 (19.64)
>30	2366 (3.56)	415 (2.5)	489 (2.94)	542 (3.26)	920 (5.54)
Silhouette at puberty					
Very thin	14,203 (21.37)	3835 (23.08)	3591 (21.61)	3484 (20.97)	3293 (19.82)
Thin	23,088 (34.74)	5753 (34.62)	5796 (34.88)	5799 (34.90)	5740 (34.54)
Medium	15,424 (23.21)	3714 (22.35)	3906 (23.51)	3910 (23.53)	3894 (23.44)
Large	10,203 (15.35)	2460 (14.81)	2474 (14.89)	2571 (15.47)	2698 (16.24)
Very large	3549 (5.34)	854 (5.14)	850 (5.12)	854 (5.14)	991 (5.96)
Education level					
<12 years	7424 (11.17)	2330 (14.02)	1765 (10.62)	1631 (9.81)	1696 (10.21)
12–14 years	35,343 (53.17)	8958 (53.91)	8963 (53.94)	8856 (53.29)	8566 (51.56)
>14 years	23,702 (35.66)	5328 (32.07)	5889 (35.44)	6131 (36.89)	6354 (38.24)
Smoking status					
Smoker	8663 (13.03)	2202 (13.25)	2130 (12.82)	2205 (13.27)	2125 (12.79)
Former smoker	21,689 (32.63)	4977 (29.95)	5398 (32.488)	5508 (33.14)	5805 (34.94)
Non-smoker	36,117 (55.34)	9437 (56.79)	9089 (54.70)	8905 (53.59)	8686 (52.27)
Physical activity (MET-hours/week)	49.10 ± 45.67	47.63 ± 46.70	48.58 ± 44.57	49.58 ± 44.72	50.62 ± 46.59
Contraceptive pill use					
Never	26,444 (39.79)	7672 (46.17)	6765 (40.71)	6111 (36.77)	5896 (35.48)
Ever	40,023 (60.21)	8944 (53.83)	9852 (59.29)	10,507 (63.23)	10,720 (64.52)
Parity					
Nulliparous	7866 (11.83)	2208 (13.29)	1956 (11.77)	1807 (10.87)	1895 (11.40)
1 or 2 children	39,322 (59.16)	9651 (58.08)	9810 (59.04)	10,012 (60.25)	9849 (59.27)
> 2 children	19,279 (29.01)	4757 (28.63)	4851 (29.19)	4799 (28.88)	4872 (29.32)
Menopausal status and recent use of menopausal hormone therapy (MHT)					
Premenopausal	37,930 (57.07)	9354 (51.05)	9418 (56.67)	9970 (60.00)	10,119 (60.90)
Menopausal and recent MHT use	8855 (13.32)	2499 (13.64)	2236 (13.46)	2183 (13.14)	2143 (12.90)
Menopausal and no recent use of MHT	17,715 (26.65)	5857 (31.96)	4482 (26.97)	4010 (24.13)	3876 (23.33)
Menopausal and no information on recent use of MHT	1967 (2.96)	614 (3.35)	482 (2.90)	455 (2.74)	479 (2.88)
Fish (g/day)	33.94 ± 23.94	20.03 ± 13.46	28.40 ± 16.21	36.52 ± 20.15	50.81 ± 30.45
Dairy products (g/day)	250.22 ± 195.43	188.35 ± 167.87	235.23 ± 179.86	264.23 ± 189.81	313.08 ± 219.15
Meat (g/day)	59.99 ± 34.22	49.32 ± 30.52	58.70 ± 32.14	63.62 ± 33.54	68.33 ± 37.36
Fat (g/day)	88.94 ± 26.91	70.01 ± 18.66	83.79 ± 20.32	93.73 ± 23.12	108.23 ± 28.65
Dioxins + DL-PCBs^a^ (pg TEQ/day min–max)	30.80 ± 12.03	20.38 ± 6.68	27.52 ± 7.13	32.91 ± 8.39	42.40 ± 12.41
NDL-PCBs^a^ (ng/day)	151.45 ± 70.16	92.83 ± 30.24	130.42 ± 35.12	162.07 ± 46.19	220.49 ± 81.57
PNNS score	3.83 ± 2.95	5.10 ± 2.57	4.40 ± 2.68	3.56 ± 2.75	2.27 ± 3.00

^a^*DL-PCB* dioxin-like polychlorinated biphenyls, *NDL-PCB* non-dioxin-like polychlorinated biphenyls.

Bold values indicate statistical significance (*p* < 0.05).

The estimated average dietary intake of total PBDEs was 41.99 ± 16.64 ng/day. The PBDEs with the highest intake was BDE 209, accounting for 61.34% of total intake (25.88 ± 12.02 ng/day), followed by BDE-47 (19.54%, 8.21 ± 4.83 ng/day) and BDE-99 (7.62%, 3.11 ± 1.19 ng/day) (supplementary Table 4). Most PBDEs congeners were strongly correlated with each other, except for BDE 183 and BDE 209, which exhibited weaker correlations with the other congeners (supplementary Table 5).

### Dietary intake of PBDEs and obesity, overweight and weight gain

All analyses showed statistically significant positive associations between dietary PBDEs intake and the risk of obesity, overweight and weight gain.

**Fig. 2:**
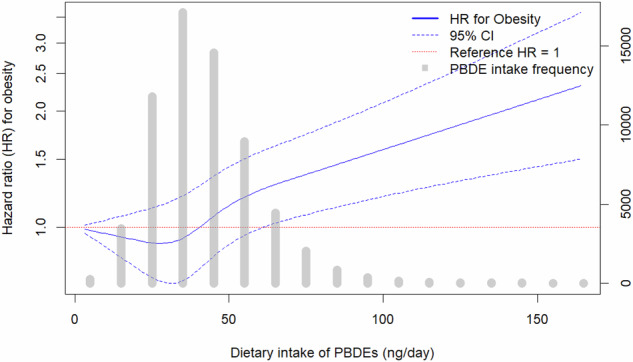
Restricted cubic spline plot of association between dietary intakes of PBDEs and risk of obesity in model 3 (*P*_non-linearity_ < 0.001). Restricted cubic splines are presented with four knots (0.05th, 0.35th, 0.65th and 0.95th). The *x*-axis shows the estimated dietary intake of PBDEs (ng/day), while the left *y*-axis (logarithmic scale) represents the HR for obesity. The grey vertical bars correspond to the frequency distribution of PBDE intake in the study population, shown on the right *y*-axis.

The quartile analyses indicated a significant positive association between dietary intake of PBDEs and obesity (model 3: HR_obesity(Q4 vs Q1)_ = 1.25 (1.15–1.37), *P*-trend_obesity_ < 0.001) (Table 2). Spline model showed a non-linearity association (*P*_non-linearity_ = 0.0014, *P*_overall_ < 0.001), with the risk of obesity initially decreasing slightly before increasing, particularly at exposure levels above 40.33 ng/day (Fig. 2).

**Table 2:** HR and 95%CI for obesity, overweight and weight gain risk according to dietary intake of PBDEs (ng/day) in E3N.

		Number of cases/ non-cases	Model 1	Model 2	Model 3
Obesity					
Splines (P _overall_ *)		5467/58,596	**0.001**	**0.001**	**0.001**
Quartiles	Q1	1095/14,923	REF	REF	REF
	Q2	1146/14,868	1.02 (0.94–1.11)	1.08 (0.99–1.17)	0.94 (0.87–1.02)
	Q3	1400/14,616	**1.25 (1.16–1.36)**	**1.36 (1.25–1.48)**	1.04 (0.96–1.13)
	Q4	1826/14,189	**1.66 (1.54–1.79)**	**1.86 (1.71–2.02)**	**1.25 (1.15–1.37)**
P trend			**<0.001**	**<0.001**	**<0.001**
Overweight					
Linear		15,874/36,849	**1.12 (1.10–1.13)**	**1.15 (1.13–1.17)**	**1.07 (1.05–1.09)**
Quartiles	Q1	3468/9716	REF	REF	REF
	Q2	3808/9371	**1.09 (1.04–1.14)**	**1.12 (1.07–1.18)**	1.03 (0.98–1.08)
	Q3	4095/9085	**1.18 (1.13–1.24)**	**1.24 (1.18–1.30)**	**1.06 (1.01–1.11)**
	Q4	4503/8677	**1.33 (1.27–1.39)**	**1.42 (1.35–1.50)**	**1.13 (1.07–1.19)**
P trend			**<0.001**	**<0.001**	**<0.001**
Weight gain					
Linear		11,257/55,210	**1.13 (1.11–1.15)**	**1.20 (1.17–1.22)**	**1.12 (1.09–1.14)**
Quartiles	Q1	2346/14,270	REF	REF	REF
	Q2	2570/14,047	1.03 (0.97–1.09)	**1.09 (1.03–1.15)**	1.03 (0.98–1.09)
	Q3	2949/13,669	**1.16 (1.10–1.22)**	**1.27 (1.20–1.34)**	**1.12 (1.06–1.19)**
	Q4	3392/13,224	**1.35 (1.28–1.43)**	**1.55 (1.46–1.65)**	**1.28 (1.21–1.37)**
*P* trend			**<0.001**	**<0.001**	**<0.001**

Model 1: adjusted for age as time scale.

Model 2: Model 1 + silhouette at puberty, smoking, alcohol, education, total energy intake without alcohol, physical activity.

Model 3: Model 2 + BMI as quintile at baseline, parity, contraceptive use and menopausal status and recent use of menopausal hormone therapy.

*P*-trend; *p*-value for linear trend estimated by fitting a semi-continuous variable based on the median value of intake of each quartile group.

Bold values indicate statistical significance (*p* < 0.05). *P-values for overall association corresponds to the test of all terms of the exposure variable (i.e. linear and non-linear terms); they are reported only when non-linear associations are detected; Restricted cubic spline plot is shown in Fig. 2.

The analysis highlighted a statistically significant positive association between dietary intake of PBDEs and the risk of overweight (model 3: HR_overweight(I SD increment)_ = 1.07 (1.05–1.09), HR_overweight(Q4 vs Q1)_ = 1.13 (1.07–1.19), *P*-trend_overweight_ < 0.001) (Table 2). There was no evidence of non-linearity for the association between dietary intake of PBDEs and overweight (*P*_non-linearity_ = 0.41).

Higher dietary intake of PBDEs were related to an increased risk of weight gain of more than 10 kg during the follow-up period (HR_weight gain(I SD increment)_ = 1.12 (1.09–1.14), HR_weight gain(Q4 vs. Q1)_ = 1.28 (1.21–1.37), *P*-trend_weight gain_ < 0.001) (Table 2). There was no evidence of non-linearity for the association between dietary intake of PBDEs and weight gain (*P*_non-linearity_ = 0.15).

### Sensitivity analyses

After further adjusting for total fat, PUFA, and n-3 PUFA intake or in the residual model, positive associations were observed between dietary intake of PBDEs and the risk of obesity, overweight, and weight gain (Table 3). These results were consistent with the main analyses after further adjustment on PNNS score or when the cases identified in the first five years of follow-up were excluded (Supplementary Tables 6 and 7). When the model was further adjusted for dietary intake of dioxins and PCBs, the results were comparable to the results obtained in the main analyses (Supplementary Table 8). In addition, the results remained similar to the main analyses when adjusting for the main food sources of PBDEs—fish, dairy products, and meats (Supplementary Table 9).

**Table 3:** HRs and 95% CIs for obesity, overweight and weight gain risk according to dietary intake of PBDEs (ng/day) in E3N adjusting for intake of total fat, PUFA, n-3 PUFA or in residual model.

		Number of cases/non-cases	M3+total fat intake	M3 + PUFA intake	M3+ n-3 PUFA	Energy residual
Obesity						
Splines (*P*_overall*_)		5467/58,596	**0.001**	**0.001**	**0.001**	**0.001**
Quartiles	Q1	1095/14,923	REF	REF	REF	REF
	Q2	1146/14,868	0.96 (0.88–1.04)	0.96 (0.88–1.04)	0.94 (0.86–1.02)	1.07 (0.99–1.17)
	Q3	1400/14,616	1.08 (0.99–1.17)	1.08 (0.99–1.17)	1.03 (0.95–1.12)	**1.14 (1.05–1.24)**
	Q4	1826/14,189	**1.35 (1.23–1.47)**	**1.35 (1.24–1.46)**	**1.24 (1.13–1.36)**	**1.39 (1.29–1.50)**
*P* trend			**<0.001**	**<0.001**	**<0.001**	**<0.001**
Overweight						
Linear		15,874/36,849	**1.06 (1.04–1.08)**	**1.06 (1.04–1.07)**	**1.06 (1.04–1.08)**	**1.05 (1.03–1.07)**
Quartiles	Q1	3468/9716	REF	REF	REF	REF
	Q2	3808/9371	1.03 (0.99–1.08)	1.03 (0.98–1.08)	1.02 (0.97–1.07)	1.03 (0.98–1.08)
	Q3	4095/9085	**1.07 (1.02–1.13)**	**1.07 (1.02–1.12)**	**1.05 (1.00–1.10)**	**1.10 (1.05–1.16)**
	Q4	4503/8677	**1.15 (1.10–1.22)**	**1.15 (1.09–1.21)**	**1.11 (1.05–1.17)**	**1.19 (1.14–1.24)**
*P* trend			**<0.001**	**<0.001**	**<0.001**	**<0.001**
Weight gain						
Linear		11,257/55,210	**1.13 (1.11–1.15)**	**1.13 (1.11–1.16)**	**1.10 (1.08–1.13)**	**1.13 (1.11–1.15)**
Quartiles	Q1	2346/14,270	REF	REF	REF	REF
	Q2	2570/14,047	1.03 (0.97–1.09)	1.03 (0.98–1.09)	1.01 (0.96–1.07)	1.06 (1.00–1.12)
	Q3	2949/13,669	**1.14 (1.08–1.21)**	**1.14 (1.08–1.21)**	**1.10 (1.04–1.17)**	**1.18 (1.12–1.25)**
	Q4	3392/13,224	**1.32 (1.24–1.41)**	**1.33 (1.26–1.41)**	**1.23 (1.16–1.32)**	**1.38 (1.31–1.46)**
*P* trend			**<0.001**	**<0.001**	**<0.001**	**<0.001**

Model 3: adjusted for age, silhouette at puberty, smoking, alcohol, education, total energy intake without alcohol, physical activity, BMI as quintile at baseline, parity, contraceptive use and menopausal status and recent use of menopausal hormone therapy.

Bold values indicate statistical significance (*p* < 0.05). *P-values for overall association corresponds to the test of all terms of the exposure variable (i.e. linear and non-linear terms); they are reported only when non-linear associations are detected.

### Subgroup analyses

These associations were further assessed according to the median follow-up time, and the positive association between dietary intake of PBDEs and three different outcomes remained unchanged (Table 4). However, when the follow-up duration exceeded median, an association was only observed between intake of PBDEs and the risk of overweight.

**Table 4:** HR and 95% CI for obesity, overweight and weight gain risk according to dietary intake PBDEs (ng/day) in E3N stratified by median follow-up for cases.

			Less than median		More than median
Obesity (24 years)					
Splines (*P_overall_)			**0.001**		**-**
Linear		5 036/59 027	**-**	431/36 793	1.07 (0.96–1.12)
Quintiles	Q1	1 005/15 013	REF	90/8 710	REF
	Q2	1 056/14 958	0.99 (0.91–1.08)	90/9 458	0.84 (0.63–1.14)
	Q3	1 280/14 736	**1.12 (1.02–1.22)**	120/9 488	1.06 (0.80–1.42)
	Q4	1 695/14 320	**1.43 (1.31–1.57)**	131/9 137	1.20 (0.88–1.63)
*P* trend			**<0.001**		0.08
Overweight (22 years)					
Linear		14,582/38,141	**1.09 (1.07–1.11)**	1292/27 081	**1.14 (1.07–1.21)**
Quintiles	Q1	3211/9973	REF	257/6 758	REF
	Q2	3492/9687	1.04 (0.99–1.09)	316/6988	1.11 (0.94–1.32)
	Q3	3763/9417	**1.09 (1.04–1.15)**	332/6815	1.17 (0.98–1.39)
	Q4	4116/9064	**1.18 (1.12–1.24)**	387/6 520	**1.38 (1.16–1.66)**
*P* trend			**<0.001**		**<0.001**
Weight gain (24 years)					
Linear		11,150/55,317	**1.15 (1.13–1.17)**	107/33,614	1.20 (0.99–1.47)
Quintiles	Q1	2327/14,289	REF	19/8 201	REF
	Q2	2549/14,068	1.05 (0.99–1.14)	21/8 621	0.94 (0.50–1.76)
	Q3	2922/13,696	**1.18 (1.11–1.25)**	27/8 704	1.13 (0.61–2.10)
	Q4	3352/13,264	**1.39 (1.31–1.47)**	40/8 088	1.63 (0.87–3.03)
*P* trend			**<0.001**		**0.05**

Model 3: adjusted for age, silhouette at puberty, smoking, alcohol, education, total energy intake without alcohol, physical activity, BMI as quintile at baseline, parity, contraceptive use and menopausal status and recent use of menopausal hormone therapy.

Bold values indicate statistical significance (*p* < 0.05). *P-values for overall association corresponds to the test of all terms of the exposure variable (i.e. linear and non-linear terms); they are reported only when non-linear associations are detected.

No statistically significant interaction was highlighted between PBDEs and BMI at baseline in relation to risks of obesity, overweight and weight gain. An interaction was observed between PBDEs and silhouette at puberty in relation to overweight (*P*_interaction _= 0.04), although the association between PBDEs and risk of overweight remained statistically significant and positive across all subgroups (data not shown).

## Discussion

The findings of this study suggest a positive linear association between dietary intake of PBDEs and the risk of overweight and weight gain. In addition, a non-linear association between the dietary intake of PBDEs and the risk of obesity was identified. The associations remained consistent when adjusting for total fat, PUFA, n-3 PUFA, as well as when adjusting for the main food sources of PBDEs (fish, dairy products, and meats). Similarly, results did not substantially change when adjusting for PCBs and dioxins intake, and when running stratified analyses based on the follow-up duration.

These results are in agreement with some previous studies that have suggested a link between PBDEs and the risk of obesity [39, 40]. Previous experimental studies have reported that PBDEs disrupt thyroid hormone signalling, inducing metabolic disturbances that can contribute to obesity [15]. PBDEs may also activate with peroxisome proliferator-activated receptor (PPAR), which are involved in lipid accumulation [41, 42]. The observed association between PBDEs and glucose metabolism further suggests that PBDEs may also increase the risk of obesity [15]. In addition, when analyses were stratified by median follow-up duration, no associations were observed for obesity or weight gain among participants with follow-up duration longer than the median. However, we cannot rule out that these differences from the main analyses were due to limited statistical power, as the number of cases included in the stratified analyses was much smaller.

Only few prospective studies have investigated the relationship between PBDEs exposure and obesity in adulthood, with inconsistent results. A Swedish study of 405 participants aged 70 years and over 80% of whom had hypertension or diabetes, found no association between plasma BDE-47 and abdominal obesity [43]. Another study in the USA, involving 468 women (with over 50% affected by obesity at baseline), found that serum BDE-47 were associated with increased BMI, while serum BDE-153 was linked to decreased BMI [22]. Of noted, the participants were elderly and over 80% had hypertension or diabetes at baseline or developed diabetes during follow-up. These health disorders could influence both the pharmacodynamics of POPs and the relationship with obesity. Furthermore, while older individuals may have higher cumulative body burdens of PBDEs due to bioaccumulation, potentially increasing their risk of obesity; age-related weight loss and change in fat distribution may also alter the association between PBDE exposure and obesity. Other studies with cross-sectional design have reporting mixed results, with one finding a positive association [21]. and others either no or inverse relationship [18–20]. However, they are unable to determine the causality link between variables and are more prone to be affected by selection bias and confounding compared to prospective studies [44].

Several factors must be considered when interpreting the association between weight change and exposure to PBDEs measured using internal biomarkers of exposure. Blood PBDE levels reflect long-term body burden, which is influenced not only by diet, but also by indoor environment, age, gender, and race/ethnicity [45]. More importantly, PBDEs are highly lipophilic, meaning they accumulate in adipose tissue over time. As a result, their blood concentrations may decrease during weight gain, as PBDEs are sequestered in the fat tissue, and increase during fat loss as they are released back into the blood [46]. These dynamic changes complicate the interpretation of the relationship between blood PBDE levels and weight changes, and potentially hide the true association between PBDEs exposure and the risk of obesity. Moreover, when studies have short follow-up or are cross-sectional, these factors could lead to a reverse causation bias, which could explain the inverse associations observed in the previous research. In the present study, we observed a positive relationship between dietary PBDEs intake and the risk of overweight, obesity and weight gain. Although dietary intake of PBDEs is highly correlated with food intake, making the analyses more prone to confounding, the models were adjusted for several confounders, and various sensitivity analyses related to diet were conducted to further assess the robustness of the results. In the E3N cohort, PBDEs intake was estimated using food frequency questionnaire at baseline combined with national food contamination data. This approach is less influenced by weight and fat tissue changes compared to the use of internal biomarkers of exposure. Moreover, the dietary information was collected at baseline, prior to the recorded weight changes. Thus, even memory bias or social desirability bias, which may lead to measurement errors, are more likely to be non-differential, leading to a potential underestimation of the true association.

Due to these differences, directly comparing our results with studies using internal biomarkers of exposure is challenging. However, both approaches have advantages and disadvantages for assessing the association and a holistic interpretation of the results should be applied.

### Strengths and limitations

Some limitations should be considered when interpreting our findings. First, the E3N cohort is composed only by middle-aged French women with higher education levels and dietary intake of PBDEs compared to the general population [10]. which may limit the generalisability. Second, PBDEs exposure at baseline may have been imperfectly estimated due to the times gap between dietary data collection (1993) and food contamination assessment (2007–2009). Nevertheless, because PBDEs have long half-lives and strong bioaccumulation in fat tissue, this discrepancy might be negligible due to the fact that food contamination levels are expected to vary only slowly along the food chain. In addition, this non-differential errors related to exposure assessment generally lead to attenuated estimates of associations in prospective studies [10, 47]. Furthermore, to allow a better determination and clarification of the associations between PBDEs intake and obesity, while accounting for exposure to other POPs exposure, our analyses were adjusted for PCBs and dioxins, which were positively associated with the risk of obesity [33]. The results indicated that the positive association between PBDEs and obesity remained statistically significant and confirming that the observed association was not driven by the co-exposure to PCBs and dioxins. However, it is also possible that other POPs may have effects on human health, potentially influencing the observed associations. In addition, even if several cofounders were included in the analyses, the associations between PBDEs intake and obesity may have been affected by other residual confounders, such as population genetic susceptibility. Finally, the present study categorised changes in weight and BMI, which may not fully capture dynamic shifts (e.g. from obesity to overweight). Weight loss is often influenced by dietary or lifestyle changes; however, because dietary data were only collected at baseline, we were unable to assess how these factors might affect the relationship between PBDEs intake and weight change.

Our study has several strengths. First, to the best of our knowledge, this study is the largest investigating the association between dietary PBDEs intakes and obesity risk and weight changes over time. Moreover, with over 20 years of follow-up available, this study allows to investigate the long-term effects of PBDEs intake strongly reducing the risk of reverse causation bias. The large sample size allowed us to conduct sensitivity and subgroup analyses, which further reinforced the results obtained from our study. The availability of body weight data collected repeatedly over follow-up strengthened our study by reducing the bias that can be caused by missing values, and allowing the accurate analyses of changes in weight over time. In the present study, we examined different outcomes, including obesity, overweight, and weight gain. These outcomes are complementary and enhance the overall value of our findings in terms of public health implications. Finally, in the E3N cohort, the richness of the data enabled adjustment for many confounders, as defined by a DAG.

## Conclusion

The present prospective study found a positive association between dietary intake of PBDEs and the risk of overweight, obesity and weight gain. These associations remained significant in multiple sensitivity and subgroup analyses confirming the robustness of these results. Further large-scale epidemiological studies from other countries, combining both external exposure and internal biomarkers, are needed to confirm our findings and to support public health decision-making in improving health policies. Further efforts, such as reinforcing public awareness and strengthening regulatory measures, are needed to reduce PBDE contamination in food and to reduce the level of exposure of the general population to PBDEs.

## Supplementary information

Supplementary material after revision

## Data Availability

Data will be made available on request.
