# Supplementary material for: Dietary intake of polybrominated diphenyl ethers (PBDEs) and the risk of obesity in the French E3N cohort
Source: Int J Obes (Lond). 2026 Jun 2;50(8):1799–808. doi: 10.1038/s41366-026-02109-z (PMC13461356; doi:10.1038/s41366-026-02109-z)
Supplement: Supplementary file 1 [file 41366_2026_2109_MOESM1_ESM.docx]

**Supplementary Figure 1. Directed acyclic graph (DAG) of the relationship between dietary exposure to PBDEs and obesity overweight and weight gain in the E3N cohort**

Exposure: Dietary exposure to PBDE

Outcome: Obesity

Covariates: Age, Alcohol, Education level, Energy intake, Menopausal status and contraceptive pill use, Parity, Physical activity, Smoking, silhouette at puberty, BMI at baseline, diet

Supplementary table 1. Baseline characteristics of the total population (N =64 063) according to cases of obesity compared with the start of follow-up

|  |  | Cases of obesity | |
| --- | --- | --- | --- |
|  | All  (N =64 063) | Cases  (N = 5 467) | Non-cases  (N = 58 596) |
| **Age (years)** | 52.41±6.66 | 51.81±6.48 | 52.47±6.68 |
| **Follow-up (years)** | 20.79±6.48 | 11.30±7.40 | 21.68±5.63 |
| **Dietary intake (ng/day)** |  |  |  |
| **Total PBDEs** | 41.76±16.46 | 45.30±17.76 | 41.43±16.30 |
| **BDE28** | 0.48±0.34 | 0.53±0.39 | 0.47±0.34 |
| **BDE47** | 8.16±4.79 | 8.99±5.38 | 8.08±4.72 |
| **BDE99** | 3.09±1.18 | 3.37±1.29 | 3.06±1.16 |
| **BDE100** | 1.64±1.03 | 1.82±1.16 | 1.63±1.02 |
| **BDE153** | 0.78±0.31 | 0.86±0.33 | 0.77±0.30 |
| **BDE154** | 0.87±0.56 | 0.97±0.63 | 0.86±0.55 |
| **BDE183** | 0.99±0.42 | 1.07±0.45 | 0.98±0.42 |
| **BDE209** | 25.75±11.91 | 27.70±12.84 | 25.57±11.81 |
|  |  |  |  |
| **BMI (kg/m^2^)** | 22.56±2.60 | 26.57 ±2.16 | 22.19 ±2.31 |
| < 18.5 | 2 254 (3.52) | 4 (0.07) | 2 250 (3.84) |
| [18.5 – 25] | 50 602 (78.99) | 1 314 (24.04) | 49 288 (84.11) |
| [25 – 30] | 11 207 (17.49) | 4 149 (75.89) | 7 058 (12.05) |
| **Silhouette at puberty** |  |  |  |
| Very thin | 13 959 (21.79) | 749 (13.70) | 13 210 (22.54) |
| Thin | 22 415 (34.99) | 1 819 (33.27) | 20 596 (35.15) |
| Medium | 14 720 (22.98) | 1 497 (27.38) | 13 223 (22.57) |
| Large | 9 677 (15.11) | 973 (17.80) | 8 704 (14.85) |
| Very large | 3 292 (5.14) | 429 (7.85) | 2 863 (4.89) |
|  |  |  |  |
| **Education level** |  |  |  |
| < 12 years | 6 977(10.89) | 820 (15.00) | 6 157 (10.51) |
| 12 to 14 years | 33 993 (53.06) | 2 962 (54.18) | 31 031 (52.96) |
| > 14 years | 23 093 (36.05) | 1 685 (30.82) | 21 408 (36.53) |
|  |  |  |  |
| **Smoking status** |  |  |  |
| Smoker | 8 388 (13.09) | 837 (15.31) | 7 551 (12.89) |
| Former smoker | 20 793 (32.46) | 1 798 (32.89) | 18 995 (32.42) |
| Non-smoker | 34 882 (54.45) | 2 832 (51.80) | 32 050 (54.70) |
|  |  |  |  |
| **Physical activity (MET-hours/day)** | 49.23± 45.55 | 46.84± 44.82 | 49.46±45.61 |
|  |  |  |  |
| **Contraceptive pill use** |  |  |  |
| Never | 25 183 (39.31) | 2 197 (40.19) | 22 986 (39.23) |
| Ever | 38 880 (60.69) | 3 270 (59.81) | 35 610 (60.77) |
| **Parity** |  |  |  |
| Nulliparous | 7 570 (11.82) | 626 (11.45) | 6 944 (11.85) |
| 1 or 2 children | 38 081 (59.44) | 3 101 (56.72) | 34 980 (59.70) |
| > 2 children | 18 412 (28.74) | 1 740 (31.83) | 16 672 (28.45) |
| **Menopausal status and recent use of menopausal hormone therapy (MHT)** |  |  |  |
| Premenopausal | 36 677 (57.25) | 3 390 (62.01) | 33 287 (56.81) |
| Menopausal and recent MHT use | 8 670(13.53) | 547 (10.01) | 8 123 (13.86 ) |
| Menopausal and no recent use of MHT | 16 816 (26.25) | 1 384 (25.32) | 15 432 (26.34) |
| Menopausal and no information on recent use of MHT | 1 900 (2.97) | 146 (2.67) | 1 754 (2.99) |
| **Total energy intake (excluding alcohol intakes kcal/day)** | 2125.98±537.33 | 2146.54±587.47 | 2124.07±532.37 |
| **Alcohol consumption (g/day)** | 11.53±13.74 | 12.06±15.28 | 11.48±13.59 |
| **Fish (g/day)** | 33.69±23.68 | 37.31±26.21 | 33.35±23.40 |
| **Dairy products (g/day)** | 249.24±195.16 | 274.29±206.80 | 246.90±193.88 |
| **Meat (g/day)** | 59.59±33.91 | 66.57±37.42 | 58.93±33.49 |
| **Fat (g/day)** | 88.62±26.64 | 92.35±29.31 | 88.27±26.35 |
| **Dioxins+DL-PCBs (pg TEQ/day)** | 30.67±11.91 | 32.42±12.68 | 30.51±11.83 |
| **NDL-PCBs (ng/day)** | 150.63±69.43 | 162.78±76.76 | 149.50±68.60 |
| **PNNS score** | 3.85±2.93 | 3.70±3.06 | 3.87±2.92 |
|  |  |  |  |
|  |  |  |  |
|  |  |  |  |

Supplementary table 2. Baseline characteristics of the total population (N =52 723) according to cases of overweight compared with the start of follow-up

|  |  | Cases of overweight | |
| --- | --- | --- | --- |
|  | All  (N =52 723) | Cases  (N = 15 874) | Non-cases  (N = 36 849) |
| **Age (years)** | 52.02±6.57 | 51.58±6.34 | 52.21±6.65 |
| **Follow-up (years)** | 17.98±8.29 | 9.50±7.01 | 21.63±5.74 |
| **Dietary exposure (ng/day)** |  |  |  |
| **Total PBDEs** | 41.31±16.23 | 42.95±16.53 | 40.61±16.05 |
| **BDE28** | 0.47±0.33 | 0.49±0.34 | 0.46±0.33 |
| **BDE47** | 8.04±4.67 | 8.42±4.82 | 7.87±4.60 |
| **BDE99** | 3.05±1.16 | 3.19±1.18 | 2.99±1.41 |
| **BDE100** | 1.61±1.01 | 1.70±1.04 | 1.58±0.99 |
| **BDE153** | 0.77±0.30 | 0.81±0.31 | 0.75±0.29 |
| **BDE154** | 0.86±0.55 | 0.90±0.56 | 0.84±0.54 |
| **BDE183** | 0.98±0.42 | 1.02±0.43 | 0.96±0.41 |
| **BDE209** | 25.54±11.79 | 26.41±11.97 | 25.16±11.69 |
|  |  |  |  |
| **BMI (kg/m^2^)** | 21.66±1.82 | 23.18 ±1.30 | 21.01 ±1.61 |
| < 18.5 | 2 254 (4.28) | 35 (0.22) | 2 219 (6.02) |
| [18.5 – 25] | 50 469 (95.72) | 15 839 (99.78) | 34 630 (93.98) |
| **Silhouette at puberty** |  |  |  |
| Very thin | 12 278 (23.29) | 3 411 (21.49) | 8 867 (24.06) |
| Thin | 18 537 (35.16) | 5 524 (34.80) | 13 013 (35.31) |
| Medium | 11 748 (22.28) | 3 603 (22.70) | 8 145 (22.10) |
| Large | 7 692 (14.59) | 2 414 (15.21) | 5 278 (14.32) |
| Very large | 2 468 (4.68) | 922 (5.81) | 1 546 (4.20) |
|  |  |  |  |
| **Education level** |  |  |  |
| < 12 years | 5 195(9.85) | 1 792 (11.29) | 3 403 (9.23) |
| 12 to 14 years | 27 757 (52.65) | 8 669 (54.61) | 19 088 (51.80) |
| > 14 years | 19 771 (37.50) | 5 413 (34.10) | 14 358 (38.96) |
|  |  |  |  |
| **Smoking status** |  |  |  |
| Smoker | 7 099 (13.46) | 2 263 (14.26) | 4 836 (13.12) |
| Former smoker | 17 126 (32.48) | 5 199 (32.75) | 11 927 (32.37) |
| Non-smoker | 28 498 (54.05) | 8 412 (52.99) | 20 086 (54.51) |
|  |  |  |  |
| **Physical activity (MET-hours/day)** | 49.36± 45.55 | 48.70± 46.90 | 49.64±44.96 |
|  |  |  |  |
| **Contraceptive pill use** |  |  |  |
| Never | 19 710 (37.38) | 5 746 (36.20) | 13 964 (37.90) |
| Ever | 33 013 (62.62) | 10 128 (63.80) | 22 885 (62.10) |
| **Parity** |  |  |  |
| Nulliparous | 6 281 (11.91) | 1 706 (10.75) | 4 575 (12.42) |
| 1 or 2 children | 32 030 (60.75) | 9 475 (59.69) | 22 555 (61.21) |
| > 2 children | 14 412 (27.34) | 4 693 (29.56) | 9 719 (26.38) |
| **Menopausal status and recent use of menopausal hormone therapy (MHT)** |  |  |  |
| Premenopausal | 31 116 (59.02) | 9 722 (61.24) | 21 394(58.06) |
| Menopausal and recent MHT use | 7 216(13.69) | 2 031 (12.79) | 5 185 (14.07) |
| Menopausal and no recent use of MHT | 12 885 (24.44) | 3 682 (23.20) | 9 203 (24.97) |
| Menopausal and no information on recent use of MHT | 1 506 (2.86) | 439 (2.77) | 1 067 (2.90) |
|  |  |  |  |
| **Total energy intake (excluding alcohol intakes kcal/day)** | 2120.74±530.22 | 2131.68±543.82 | 2116.03±524.19 |
| **Alcohol consumption (g/day)** | 11.44±13.52 | 12.31±14.32 | 11.06±13.14 |
| **Fish (g/day)** | 33.04±23.08 | 34.63±23.99 | 32.35±22.64 |
| **Dairy products (g/day)** | 245.48±192.16 | 257.05±197.87 | 240.50±189.44 |
| **Meat (g/day)** | 58.59±33.37 | 62.93±34.89 | 56.72±32.51 |
| **Fat (g/day)** | 88.07±26.26 | 90.09±26.88 | 87.20±25.95 |
| **Dioxins+DL-PCBs (pg TEQ/day)** | 30.43±11.78 | 31.19±11.93 | 30.10±11.70 |
| **NDL-PCBs (ng/day)** | 148.80±67.91 | 154.32±69.87 | 146.42±66.9 |
| **PNNS score** | 3.86±2.91 | 3.4±2.94 | 3.92±2.89 |

Supplementary table 3. Baseline characteristics of the total population (N =66 467) according to cases of weight gain compared with the start of follow-up

|  |  | Cases of weight gain >= 10kg compared to baseline | |
| --- | --- | --- | --- |
|  | All  (N = 66 467) | Cases  (N = 11 257) | Non-cases  (N = 55 210) |
| **Age (years)** | 52.47±6.68 | 50.38±5.92 | 52.90±6.74 |
| **Follow-up (years)** | 20.00±6.62 | 12.92±5.76 | 21.43±5.83 |
| **Dietary exposure (ng/day)** |  |  |  |
| **Total PBDEs** | 41.99±16.64 | 44.44±17.30 | 41.49±16.46 |
| **BDE28** | 0.48±0.34 | 0.51±0.37 | 0.48±0.34 |
| **BDE47** | 8.21±4.83 | 8.72±5.13 | 8.11±4.77 |
| **BDE99** | 3.11±1.19 | 3.31±1.25 | 3.06±1.18 |
| **BDE100** | 1.65±1.04 | 1.76±1.11 | 1.63±1.03 |
| **BDE153** | 0.78±0.31 | 0.84±0.33 | 0.77±0.31 |
| **BDE154** | 0.88±0.57 | 0.94±0.60 | 0.87±0.56 |
| **BDE183** | 0.99±0.43 | 1.06±0.45 | 0.98±0.42 |
| **BDE209** | 25.88±12.02 | 27.29±12.45 | 25.59±11.91 |
|  |  |  |  |
| **BMI (kg/m^2^)** | 22.94 ±3.25 | 24.00 ±3.60 | 22.72 ±3.13 |
| < 18.5 | 2 254 (3.39) | 220 (1.95) | 2 034 (3.68) |
| [18.5 – 25] | 50 602 (76.13) | 7 532 (66.91) | 43 070 (78.01) |
| [25 – 30] | 11 245 (16.92) | 2 770 (24.61) | 8 475 (15.35) |
| >30 | 2 366 (3.56) | 735 (6.53) | 1 631 (2.95) |
| **Silhouette at puberty** |  |  |  |
| Very thin | 14 203 (21.37) | 2 358 (20.95) | 11 845 (21.45) |
| Thin | 23 088 (34.74) | 3 892 (34.57) | 19 196 (34.77) |
| Medium | 15 424 (23.21) | 2 658 (23.61) | 12 766 (23.12) |
| Large | 10 203 (15.35) | 1 686 (14.98) | 8 517 (15.43) |
| Very large | 3 549 (5.34) | 663 (5.89) | 2 886 (5.23) |
|  |  |  |  |
| **Education level** |  |  |  |
| < 12 years | 7 424 (11.17) | 1 351 (12.00) | 6 071 (11.00) |
| 12 to 14 years | 35 343 (53.17) | 6 029 (53.56) | 29 314 (53.10) |
| > 14 years | 23 702 (35.66) | 3 877 (34.44) | 19 825 (35.91) |
|  |  |  |  |
| **Smoking status** |  |  |  |
| Smoker | 8 663 (13.03) | 1 893 (16.82) | 6 769 (12.26) |
| Former smoker | 21 689 (32.63) | 3 684 (32.73) | 18 004 (32.61) |
| Non-smoker | 36 117 (55.34) | 5 680 (50.46) | 30 437 (55.13) |
|  |  |  |  |
| **Physical activity (MET-hours/day)** | 49.10±45.67) | 46.93± 44.84 | 49.55±45.82 |
|  |  |  |  |
| **Contraceptive pill use** |  |  |  |
| Never | 26 444 (39.79) | 3 760 (33.40) | 22 684 (41.09) |
| Ever | 40 023 (60.21) | 7 497 (66.60) | 32 526 (58.91) |
| **Parity** |  |  |  |
| Nulliparous | 7 866 (11.83) | 1 257 (11.17) | 6 609 (11.977) |
| 1 or 2 children | 39 322 (59.16) | 6 788 (60.30) | 32 534 (58.93) |
| > 2 children | 19 279 (29.01) | 3 212 (28.53) | 16 067 (29.10) |
| **Menopausal status and recent use of menopausal hormone therapy (MHT)** |  |  |  |
| Premenopausal | 37 930 (57.07) | 7 553 (67.10) | 30 377 (55.02) |
| Menopausal and recent MHT use | 8 855 (13.32) | 1 198 (10.64) | 7 657 (13.87) |
| Menopausal and no recent use of MHT | 17 715 (26.65) | 2 245 (19.94) | 15 470 (28.02) |
| Menopausal and no information on recent use of MHT | 1 967 (2.96) | 261 (2.32) | 1 706 (3.09) |
| **Total energy intake (excluding alcohol intakes kcal/day)** | 2130.83±542.01 | 2145.49±570.08 | 2127.85±536.06 |
| **Alcohol consumption (g/day)** | 11.53±13.84 | 12.34±15.04 | 11.34±13.53 |
| **Fish (g/day)** | 33.94±23.94 | 35.90±25.37 | 33.54±23.62 |
| **Dairy products (g/day)** | 250.22±195.43 | 261.00±202.00 | 248.03±193.99 |
| **Meat (g/day)** | 59.99±34.22 | 64.72±36.52 | 59.02±33.64 |
| **Fat (g/day)** | 88.94±26.91 | 91.78±28.42 | 88.36±26.55 |
| **Dioxins+DL-PCBs (pg TEQ/day)** | 30.80±12.03 | 32.03±12.38 | 30.55±11.93 |
| **NDL-PCBs (ng/day)** | 151.45±70.16 | 158.96±73.44 | 149.92±69.38 |
| **PNNS score** | 3.83±2.95 | 3.58±3.04 | 3.89±2.93 |

# Supplementary table 4. Proportion of each PBDE congener to the total PBDEs dietary intake in the E3N cohort (N=66 467).

| **Congener** | **Proportion** |
| --- | --- |
| BDE-28 | 1.13 |
| BDE-47 | 19.54 |
| BDE-99 | 7.62 |
| BDE-100 | 3.92 |
| BDE-153 | 1.91 |
| BDE-154 | 2.08 |
| BDE-183 | 2.44 |
| BDE-209 | 61.34 |

Supplementary table 5: Spearman rank correlations coefficients between dietary intake of each PBDE congeners in the E3N cohort (N=66 467).

|  | **BDE-28** | **BDE-47** | **BDE-99** | **BDE-100** | **BDE-153** | **BDE-154** | **BDE-183** | **BDE-209** |
| --- | --- | --- | --- | --- | --- | --- | --- | --- |
| **BDE-28** |  |  |  |  |  |  |  |  |
| **BDE-47** | 0.98209 |  |  |  |  |  |  |  |
| **BDE-99** | 0.70556 | 0.81606 |  |  |  |  |  |  |
| **BDE-100** | 0.99112 | 0.99544 | 0.77543 |  |  |  |  |  |
| **BDE-153** | 0.64530 | 0.73979 | 0.90480 | 0.71014 |  |  |  |  |
| **BDE-154** | 0.98748 | 0.98506 | 0.75419 | 0.99091 | 0.72877 |  |  |  |
| **BDE-183** | 0.23086 | 0.34280 | 0.63044 | 0.29896 | 0.76455 | 0.31675 |  |  |
| **BDE-209** | 0.23147 | 0.32374 | 0.55834 | 0.27784 | 0.58356 | 0.27693 | 0.70203 |  |

All p-values are <0.0001

Supplementary table 6 HRs and 95%CIs for obesity overweight and weight gain risk according to dietary intakes of PBDEs (ng/d) in E3N after adjusted on PNNS score

|  | Obesity | | | Overweight | | | Weight gain | | |
| --- | --- | --- | --- | --- | --- | --- | --- | --- | --- |
| Splines (P _overall_) | 0.001 | | |  | | - |  | | - |
| Linear |  | 5 467/58 596 | **-** | 15 874/36 849 | **1.08 (1.07-1.10)** | | 11 257/55 210 | **1.14 (1.12-1.17)** | |
| Quintiles | Q1 | 1 095/14 923 | REF | 3 468/9 716 | REF | | 2 346/14 270 | REF | |
|  | Q2 | 1 146/14 868 | 0.98 (0.90-1.07) | 3 808/9 371 | 1.05 (0.99-1.10) | | 2 570/14 047 | 1.05 (0.99-1.12) | |
|  | Q3 | 1 400/14 616 | **1.11 (1.02-1.21)** | 4 095/9 085 | **1.09 (1.04-1.15)** | | 2 949/13 669 | **1.17 (1.11-1.24)** | |
|  | Q4 | 1 826/14 189 | **1.41 (1.29-1.53)** | 4 503/8 677 | **1.18 (1.12-1.24)** | | 3 392/13 224 | **1.37 (1.29-1.46)** | |
| P trend |  |  | **<0.001** |  | **<0.001** | |  | **<0.001** | |

Model 3: adjusted for age silhouette at puberty smoking alcohol education total energy intake without alcohol physical activity BMI as quintile at baseline parity contraceptive use and menopausal status and recent use of menopausal hormone therapy and PNNS score

Supplementary table 7 HRs and 95%CIs for obesity overweight and weight gain risk according to dietary intakes of PBDEs (ng/d) in E3N after excluding the cases identified in the first five years of follow-up

|  | Obesity | | | Overweight | | Weight gain | |
| --- | --- | --- | --- | --- | --- | --- | --- |
| Linear |  | 3 948/56 917 | **1.14 (1.10-1.18)** | 10 103/35 668 | **1.09 (1.06-1.11)** | 10 106/53 446 | **1.14 (1.12-1.17)** |
| Quintiles | Q1 | 806/14 411 | REF | 2 204/9 239 | REF | 2 109/13 779 | REF |
|  | Q2 | 849/14 366 | 0.98 (0.89-1.08) | 2 403/9 040 | 1.03 (0.97-1.10) | 2 327/13 561 | 1.06 (1.00-1.13) |
|  | Q3 | 1 008/14 209 | 1.07 (0.97-1.18) | 2 619/8 824 | **1.09 (1.03-1.16)** | 2 682/13 206 | **1.19 (1.12-1.27)** |
|  | Q4 | 1 285/13 931 | **1.36 (1.23-1.51)** | 2 877/8 565 | **1.20 (1.12-1.28)** | 2 988/12 900 | **1.36 (1.27-1.45)** |
| P trend |  |  | **<0.001** |  | **<0.001** |  | **<0.001** |

Model 3: adjusted for age silhouette at puberty smoking alcohol education total energy intake without alcohol physical activity BMI as quintile at baseline parity contraceptive use and menopausal status and recent use of menopausal hormone therapy

Supplementary table 8 HRs and 95%CIs for obesity overweight and weight gain risk according to dietary intakes of PBDEs (ng/d) in E3N adjusting for dietary exposure to dioxins and PCBs

|  |  | Number of cases/non-cases | M3+ dixions+DL-PCBs | | M3+ NDL-PCBs |
| --- | --- | --- | --- | --- | --- |
| Obesity | | | |  |  |
| Splines (P _overall_) |  | 5 467/58 596 | 0.01 | | 0.01 |
| Quartiles | Q1 | 1 095/14 923 | REF | | REF |
|  | Q2 | 1 146/14 868 | 0.95 (0.87-1.03) | | 0.95 (0.87-1.03) |
|  | Q3 | 1 400/14 616 | 1.06 (0.97-1.15) | | 1.05 (0.96-1.15) |
|  | Q4 | 1 826/14 189 | **1.29 (1.16-1.42)** | | **1.27 (1.15-1.41)** |
| P trend |  |  | **<0.001** | | **<0.001** |
| P _Dioxin or PCB (quartile)_ | |  | **<0.001** | | **<0.001** |
| Overweight | | | |  |  |
| Linear |  | 15 874/36 849 | **1.09 (1.07-1.12)** | | **1.09 (1.06-1.11)** |
| Quartiles | Q1 | 3 468/9 716 | REF | | REF |
|  | Q2 | 3 808/9 371 | 1.04 (0.99-1.09) | | 1.03 (0.98-1.08) |
|  | Q3 | 4 095/9 085 | **1.08 (1.03-1.14)** | | **1.07 (1.02-1.13)** |
|  | Q4 | 4 503/8 677 | **1.16 (1.10-1.23)** | | **1.14 (1.07-1.22)** |
| P trend |  |  | **<0.001** | | **<0.001** |
| P _Dioxin or PCB (quartile)_ | |  | 0.09 | | **0.01** |
| Weight gain | | | |  |  |
| Linear |  | 11 257/55 210 | **1.12 (1.09-1.15)** | | **1.12 (1.09-1.15)** |
| Quartiles | Q1 | 2 346/14 270 | REF | | REF |
|  | Q2 | 2 570/14 047 | 1.02 (0.96-1.08) | | 1.02 (0.96-1.08) |
|  | Q3 | 2 949/13 669 | **1.12 (1.05-1.19)** | | **1.11 (1.04-1.18)** |
|  | Q4 | 3 392/13 224 | **1.27 (1.18-1.36)** | | **1.25 (1.16-1.34)** |
| P trend |  |  | **<0.001** | | **<0.001** |
| P _Dioxin or PCB (quartile)_ | |  | **<0.001** | | **<0.001** |

Model 3: adjusted for age silhouette at puberty smoking alcohol education total energy intake without alcohol physical activity BMI as quintile at baseline parity contraceptive use and menopausal status and recent use of menopausal hormone therapy

Supplementary table 9 HRs and 95%CIs for obesity overweight and weight gain risk according to dietary intakes of PBDEs (ng/d) in E3N after adjusted for fish dairy products and meat intakes

| PBDE | Obesity | | | | Overweight | | Weight gain | |
| --- | --- | --- | --- | --- | --- | --- | --- | --- |
| Splines (P _overall_) |  | | | 0.001 |  | |  | |
| Linear | |  | 5 467/58 596 | - | 15 874/36 849 | 1.06 (1.04-1.09) | 15 874/36 849 | 1.10 (1.08-1.13) |
| Quintiles | | Q1 | 1 095/14 923 |  | 3 501/9 680 |  | 3 501/9 680 |  |
|  | | Q2 | 1 146/14 868 | 0.93 (0.86-1.01) | 3 805/9 376 | 1.01 (0.97-1.06) | 3 805/9 376 | 1.00 (0.95-1.06) |
|  | | Q3 | 1 400/14 616 | 1.03 (0.95-1.12) | 4 076/9 105 | 1.04 (0.99-1.10) | 4 076/9 105 | 1.09 (1.02-1.15) |
|  | | Q4 | 1 826/14 189 | 1.25 (1.14-1.38) | 4 492/8 688 | 1.10 (1.04-1.17) | 4 492/8 688 | 1.22 (1.14-1.31) |
| P trend | |  |  | <0.001 |  | 0.0004 |  | <0.001 |

Model 3: adjusted for age silhouette at puberty smoking alcohol education total energy intake without alcohol physical activity BMI as quintile at baseline parity contraceptive use and menopausal status and recent use of menopausal hormone therapy and fish dairy products and meat intakes
